# Green and Generous: Virtue Signaling Environmentalism and Community-Mindedness from an Evolutionary Perspective

**DOI:** 10.1177/14747049261423756

**Published:** 2026-02-20

**Authors:** Maryanne L. Fisher, Hidenori Komatsu, Hiromi Kubota, Nobuyuki Tanaka, Mariah Griffin, Glenn Geher

**Affiliations:** 1 Department of Psychology, 3690 Saint Mary’s University, Halifax, NS, Canada; 2 Grid Innovation Research Laboratory, 580868 Central Research Institute of Electric Power Industry, Yokosuka, Kanagawa, Japan; 3 Sustainable System Research Laboratory, 92141 Central Research Institute of Electric Power Industry, Abiko, Chiba, Japan; 4 Department of Psychology, 14821 State University of New York at New Paltz, New Paltz, NY, USA

**Keywords:** sex differences, Big Five, cross-cultural, group dynamics, interpersonal relationships

## Abstract

We explore the self-reported propensity for virtue signaling of environmentalism and community-minded messages. Evolutionary psychologists have not paid much attention to virtue signaling, although it has implications for social relationships, in-group/out-group dynamics, status, mate choice, and kinship. We tested three hypotheses regarding sex differences in samples (*N* = 20,423) obtained in 2020 from Canada, Japan, and the USA. First, across all samples, we hypothesized that both sexes use environmentalism and community-mindedness to engage in virtue signaling, which was supported. Second, we hypothesized that conspicuous ethical consumption, as a form of virtue signaling, is performed more by women than by men, which was not supported. Contrary to our prediction, men reported higher engagement in conspicuous ethical consumption, suggesting that status motives may have a strong role in their virtue signaling behaviors. Third, we hypothesized that known sex differences in the Big Five personality traits are linked to virtue signaling. That is, typically, women are reported to score higher than men in neuroticism, extroversion, agreeableness, and conscientiousness, while men score higher on openness than women. Our findings partially support this pattern. Last, we report on intercultural differences. We close with a discussion of the usefulness of studying virtue signaling from an evolutionary perspective.

## Introduction

Virtue signaling, the act of publicly expressing moral values to gain social approval, can be understood through an evolutionary lens as a behavior that enhances fitness by solving adaptive problems. Virtue signaling may have evolved as a mechanism to facilitate mate selection, in-group cooperation, and status competition. For example, signaling moral virtues such as generosity, honesty, or environmental stewardship could enhance an individual’s social standing, making them more attractive as cooperative partners or mates (Miller, 2007; Zahavi, 1975). These behaviors may also serve to strengthen group cohesion by signaling adherence to shared norms and values, thereby promoting trust and cooperation within the group (Smith & Bird, 2005).

Virtue signaling can involve actions, like posting on social media, that may be of low cost and primarily aimed at gaining recognition, while others, such as attending protests, can involve significant personal effort and risk. These actions may therefore serve not only as signals of virtue but also as contributions to environmental or social causes (e.g., Plows, 2008). The core of virtue signaling may be “performative but ultimately empty displays of moral goodness” (Pinder-Amaker & Wadsworth, 2023), but it is important to recognize that not all actions labeled as virtue signaling are devoid of genuine impact.

The primary motivation behind this behavior, when performed as virtue signaling, is to gain recognition and admiration from others for appearing virtuous often without putting in any real effort (see Bartholomew 2015a, 2015b). The idea of virtue signaling has become part of the popular vernacular and has gained some attention in evolutionary psychology, particularly through Miller's (2007) work, but it remains underexplored compared to other topics in the field. This lack of attention is intriguing because it is directly tied to social manipulation, status, and impression management. Moreover, virtue signaling may be used to gain benefits that have repercussions for social living; someone may signal their morals to obtain social, sexual, or status advantages (Miller, 2007). For example, Miller (2007) proposes that virtues may have evolved in both men and women via mate choice, such that they are linked to signaling genetic quality, parenting ability, or other features that are relevant to mating (e.g., kindness, honesty, intelligence, and agreeableness, all of which are commonly listed as long-term mate preferences, Buss, 1989).

On this basis, sex differences in virtue signaling can be understood through the lens of sexual selection and differential reproductive strategies. Women, historically tasked with caregiving and social cohesion, may signal traits like agreeableness and conscientiousness to demonstrate their suitability as cooperative partners and caregivers (Buss, 1989; Eagly & Wood, 1991). Men, on the other hand, may engage in costly displays of status and resource acquisition, aligning with their roles as providers and protectors (Griskevicius et al., 2010). These sex-specific strategies are consistent with costly signaling theory, which posits that individuals engage in resource-intensive behaviors to signal their fitness and value to others (Zahavi, 1975).

Beyond behaviors, personality itself may also function as a signal, offering curated information to others that may differ from one’s actual personality. Komatsu et al. (2024) reported that one’s signaled personality, and whether opposite-sex perceivers believe it to be similar or not to their own personality, can influence mate choice, even for pairs who just met and conversed online for 30 minutes with their faces hidden and with their voices transformed into a neutral pitch.

Extending beyond individual traits, cultural differences in virtue signaling may reflect local adaptive pressures. For example, in collectivist societies like Japan, virtue signaling may emphasize group harmony and social conformity, while in individualistic cultures like the USA, it may focus on personal achievement and status enhancement. These cultural variations highlight the flexibility of virtue signaling as an adaptive strategy that responds to ecological and social contexts.

### The Functions of Virtue Signaling

In general, virtue signaling is thought to have originated from an article by Bartholomew (2015a; see also Bartholomew 2015b), where he proposed that it serves as a camouflage, whereby one can emphasize publicly what they hate or oppose, for example. It distracts others into thinking the signaler is unconsciously advertising, in an accurate manner, how strongly they are moral, good, or virtuous. According to Bartholomew (2015a, 2015b), the signaler’s goal is to demonstrate kindness and intelligence yet avoid getting into facts or evidence, without having to actually do anything. It is an easy, even lazy, way to signal one’s positive qualities to others.

This halo of negative perception surrounding those who act virtuously has been documented for those who engage in prosocial behavior. Berman and Silver (2022) outlined that people act prosocially to gain reputational benefit when people perceive them as being generous. However, prosocial individuals are often viewed with suspicion or considered “disingenuous braggarts, empty virtue-signalers, or holier-than-thou hypocrites” (Berman & Silver, 2022, p. 102). But this perception of being disingenuous is highly context dependent. Several factors, including the role of potential observers, the setting in which the signal is displayed, and the relative or symbolic costs associated with the signal, influence whether it is perceived as genuine or self-serving. For instance, (Engeler & Raihani, 2024) argued that the presence of observers can amplify the reputational benefits of prosocial behaviors, as individuals are more likely to engage in uncalculated, spontaneous actions when they know they are being watched. These uncalculated actions are often perceived as more trustworthy because they signal genuine prosocial intent rather than strategic self-interest (Engeler & Raihani, 2024). Meanwhile, when the costs to the signal are perceived as minimal or the actions appear overly calculated, virtue signaling can backfire, leading to negative perceptions. Observers may view such signals as empty displays of moral goodness, aimed solely at gaining social approval without genuine commitment to the cause (Raihani & Power, 2021). For example, public displays of environmentalism, such as purchasing organic food or driving an electric car, may be dismissed as performative if the individual does not demonstrate consistent behavior across other domains.

While virtue signaling is often criticized for being disingenuous, evidence suggests it may have positive effects. One such effect is conveying confidence and information about the number of people who share a particular position (Levy, 2020). That is, one purposely avoids putting forth a position that is disagreeable or in the minority, as it will diminish one’s social reputation. Thus, members in the social group may learn about group norms and expectations by paying attention to what information is signaled. One way this occurs is through conformity information, which refers to the idea that people are more likely to conform to a particular behavior or belief if they believe others are doing the same (Cialdini & Goldstein, 2004). When individuals engage in virtue signaling, they inform their social group about acceptable or desirable beliefs and behaviors.

Further, it is part of social comparison, as one is proposing that they are virtuous against the backdrop of others’ behavior. Festinger (1954) suggested that people have an inherent need to evaluate themselves, and when objective measures are not available, people opt to compare themselves with others to gauge their standing. Part of social comparison may include individuals attempting to differentiate themselves concerning their preferences, beliefs, and actions, enabling them to gain social status and approval. Niemietz (2014) reviewed how people might benefit from standing out, whether for their preferences, actions, or beliefs. For example, someone might publicly participate in a community cleanup event, not only to contribute to environmental improvement but also to signal their commitment to community values and environmental stewardship. Such actions allow individuals to differentiate themselves positively within their social group, gaining recognition and approval for their visible contributions to shared goals.

Indeed, one’s need for uniqueness is important in many domains. Tian et al. (2001) examined this need in consumer behavior. They defined it as “the trait of pursuing differentness relative to others through the acquisition, utilization and disposition of consumer goods to enhance one’s self-image and social image” (p. 52). They suggested it is composed of three dimensions. First, people seek out choices that differ from others while being aware that others would presumably consider them good choices. Second, they may consume goods that deviate from group norms and consequently incur some group disapproval, and third, they lose interest in commonplace items because they are not unique.

Within the context of virtue signaling, we propose that individuals promote their unique morals by socially comparing themselves against others who hold less moral or unpopular views. In effect, they stand out for being uniquely moral and virtuous compared to others in their social network. Social comparison entails individuals responding to the actions of others, such that they can effectively socially integrate and adjust to what is situationally appropriate (Bearden & Rose, 1990) or what sort of virtue signaling would be needed to be noticed by others.

It is relevant to note that virtue signaling is different from moral grandstanding. As introduced by Tosi and Warmke (2016), moral grandstanding refers to the use of moral talk for status-seeking. Hence, the motivation is to enhance one’s social status or rank, which differs from one of the goals of virtue signaling, which is signaling moral respectability. Levy (2020) echoed this sentiment; while moral grandstanding is undoubtedly linked to the concept of virtue signaling, virtue signaling goes further by connecting signaling theory and social displays. Pinder-Amaker and Wadsworth (2023) argued that virtue signaling is a way to demonstrate publicly, even if just in words, one’s commitment to specific values, which goes beyond status enhancement. Further, McCosker and Johns (2014) proposed that virtue signaling may allow individuals to signal their adherence to socially approved norms and values, again differing from grandstanding. For clarity, here, we refer to the signaling of one’s morals to improve their status and perceived value as virtue signaling.

### Evolutionary Psychology of Virtue Signaling

Virtue signaling has far-reaching implications for social relationships, in-group/out-group dynamics, status, mate choice, and kinship. For example, it may promote feelings of trust and credibility within social relationships, as individuals who signal prosocial behaviors are often perceived as more trustworthy and cooperative (Barclay & Barker, 2020; Griskevicius et al., 2010). It also plays a role in in-group/out-group behavior, as signaling adherence to group norms can strengthen bonds within the in-group while differentiating oneself from out-groups (Winegard, 2018). Additionally, public demonstrations of virtue can elevate an individual’s social status, signaling traits like generosity and trustworthiness that are desirable in both social and mating contexts (Fraser, 2010; Miller, 2007).

Using an experimental paradigm, Griskevicius et al. (2010) established that individuals who are motivated by status choose environmentally minded products (i.e., “green” products) over more luxurious but non-environmentally minded products. They argued that selecting such items signals altruism by showing that one is willing and able to incur costs to themselves for the benefit of group members. Interestingly, triggering status motives led to a stronger interest in purchasing green products in public but not private venues and when the green products cost more than nongreen items.

Costly signaling theory provides the theoretical foundation for understanding virtue signaling. Costly signaling posits that individuals engage in behaviors that are effortful, resource intensive, or risky to demonstrate their fitness or value to others (McAndrew, 2021; Zahavi, 1975). These signals are reliable because their cost ensures that only individuals with the necessary resources or traits can afford to perform them. Virtue signaling can be viewed as a specific application of costly signaling, focusing on moral and prosocial behaviors that signal desirable traits such as trustworthiness, generosity, and adherence to group norms. For example, conspicuous ethical consumption, such as purchasing expensive eco-friendly products, serves as a costly signal of both wealth and moral commitment (Griskevicius et al., 2010). Thus, costly signaling is a broader evolutionary framework, while virtue signaling is a narrower construct that applies these principles to moral and social behaviors. However, the two constructs are deeply connected, and virtue signaling is best understood as an extension of costly signaling theory (e.g., Griskevicius et al., 2010).

One element of virtue signaling is a public assertion regarding one’s adherence to socially valued norms and principles. Engaging in such activity can directly lead to securing positive relationships and social approval from others (Jordan et al., 2016). It is advantageous for individuals to publicly punish those who are selfish to signal that they, themselves, are not selfish. Thus, those who punish others are trusted more and behave in a more trustworthy manner than those who do not punish others, particularly if they do not have the opportunity to help directly. That is, when a punisher has the opportunity to help (and hence alter the behavior so punishment would not be warranted), they are less likely to punish, and if they do enact punishment, it is seen as a weaker signal of trustworthiness (Jordan et al., 2016).

Virtue signaling also influences in-group and out-group dynamics. Those who use virtue signals affirm their allegiance toward their group while differentiating themselves from the out-group (Winegard, 2018). For example, one’s endorsement of a specific moral or political belief may signal affiliation with a certain in-group and strengthen bonds within that group while creating distance from those who do not share the same values.

In addition, virtue signaling is a significant way for individuals to influence their status within a social hierarchy. Public demonstrations of virtue may elevate an individual’s social standing, as they are perceived as more altruistic, trustworthy, and cooperative (Miller, 2019). This increased status can have tangible benefits, such as improved access to resources or potential mates. Indeed, Griskevicious et al. (2010) pointed out that virtue signaling can indicate desirable qualities in a potential mating partner. Demonstrations of generosity, kindness, or fairness can signal that an individual will likely be a good partner or parent, influencing others’ perceptions of attractiveness.

Recent studies further support and extend the conclusions of Griskevicius et al. (2010) by demonstrating that public displays of pro-environmental behavior and altruism consistently enhance perceptions of attractiveness. For example, Palomo-Vélez et al. (2020) found that individuals engaging in conspicuous conservation are regarded as more attractive, particularly for long-term relationships. Farrelly and King (2019) showed that altruism is a desirable trait across sex and relationship contexts, indicating its importance in mate selection. Borau et al. (2021) found that men’s visible green consumption signals long-term commitment, and Farrelly and Bhogal (2021) confirmed that individuals increase pro-environmental behaviors in the presence of potential mates. Collectively, these findings demonstrate that pro-environmental and altruistic signaling reliably conveys commitment, trustworthiness, and desirability as a partner.

### Virtue Signaling of Environmental and Community-Oriented Messages

Virtue signaling has become increasingly prevalent in environmental discourses such that on an individual level, virtue signaling frequently manifests in pro-environmental behaviors. Wang et al. (2016) delved into this dynamic, exploring how individuals may signal their “green” identity and commitment to environmental causes through actions such as recycling or choosing sustainable products. These behaviors not only contribute to environmental conservation but also communicate the individual’s alignment with socially valued environmental norms. For example, Wang et al. (2020) reviewed that the act of purchasing organic food can be a form of virtue signaling, demonstrating support for environmental sustainability and health consciousness. This decision communicates a message about personal values and priorities to others.

Virtue signaling behaviors, however, exist on a spectrum, ranging from low-cost, performative actions to more effortful displays that serve as reliable signals of commitment to prosocial values. While some actions, such as posting on social media or wearing symbolic items, may require minimal effort and primarily aim to gain social approval, others, such as conspicuous ethical consumption, involve significant personal investment. For example, purchasing expensive eco-friendly products or participating in community cleanup events may require financial resources, time, or inconvenience. These more effortful behaviors align with costly signaling theory (Zahavi, 1975), which posits that the credibility of a signal is enhanced by the effort or cost it involves. By incurring these costs, individuals demonstrate their commitment to the values they signal, thereby reinforcing their social standing and trustworthiness within their group (Barclay & Barker, 2020; Griskevicius et al., 2010).

Environmental movements are often connected to one’s community. Websites tout the benefits of involvement in local, community-based efforts to improve one’s environmental influence (e.g., https://greenisthenewblack.com/how-to-get-involved-with-community-environmental-efforts/). Initiatives, such as clean-air movements, to improve one’s environment may occur at the community level and, indeed, allow individuals to contribute to ongoing monitoring of specific concerns (Griswold et al., 2022). Community orientation is also evident in popular “buy local” movements, such that goods are transported shorter distances, leading to less pollution, for example. Murphy (2016) argued that this focus on local goods is tied to conspicuous consumption used to signal one’s status.

Moreover, environment-based virtue signaling is tied to social relationships that indicate cooperation with others. Individuals generally strive to showcase their cooperative intent by adopting pro-environmental stances (Barclay & Barker, 2020). People donate more to environmental charities when their donations are public versus anonymous, but especially when they want to gain reputational benefit. Using a cooperative game paradigm, Barclay and Barker (2020) reported that those who donated larger amounts were selected more often as partners. However, the critical point is that those who donated larger amounts were *actually more* cooperative, meaning environmental donations reliably informed participants’ future cooperativeness.

### Virtue Signaling Ethical Consumption

When consumers choose products with explicit environmental messages, such as those labeled as “organic,” “recycled,” or “sustainably sourced,” they are not just buying a product but also buying into an identity they project to others. These choices serve as visible signals of environmental values and commitments, whereby individuals communicate that they prioritize the environment and are willing to make personal consumption choices that reflect this priority. However, White et al. (2019) outlined the existence of a paradox: while individuals, especially millennials, declare that they want brands that have purpose and environmental sustainability, few purchase such goods. Thus, it could be the case that those who do so stand out for engaging in such consumption.

Carfagna et al. (2014) contended that ethical consumers, often seen as niche or fringe elements, are increasingly becoming part of mainstream consumer practices and products. This shift is fueled mainly by the desire to signal ethical stances and environmental consciousness to others. As White et al. (2019) noted, though, actual consumption patterns might lag behind declarations of intentions. Purchasing such ethical goods may also be used to signal prosociality. For example, Luomala et al. (2020) reported that consumers who favor organic food are considered highly prosocial and that this belief extends to how those consumers are treated socially. Thus, people might purchase organic food, among other goods, to virtue signal.

### Sex Differences in Virtue Signaling

Sex differences in virtue signaling may be rooted in both evolutionary and social factors. Women are often more attuned to social norms and expectations, which may enhance their engagement in prosocial and community-oriented behaviors (Eagly & Wood, 1991). This tendency aligns with the caregiving and social cohesion roles historically associated with women, as well as their higher levels of agreeableness and conscientiousness (Costa et al., 2001). From an evolutionary stance, these traits may be associated with good parenting skills, along with traits such as reliability and nurturance, and are often evaluated during mate selection because they indicate the ability to invest in offspring and maintain cooperative relationships (Buss, 1989).

In contrast, men may engage in virtue signaling to enhance their status and competitiveness, aligning with costly signaling theory (Griskevicius et al., 2010). Costly signaling theory posits that individuals engage in behaviors that are effortful or resource intensive to demonstrate their fitness or value to others. For men, this process often involves public displays of generosity or heroism, which serve as costly signals of their ability to bear resources and provide benefits to others, thereby enhancing their attractiveness as mates (Miller, 2007). For example, research in rural Western China found that men were more likely to participate in high-cost, infrequent public displays, such as distant pilgrimages, which enhanced their reputations for prosociality and status (Cai et al., 2023). These findings suggest that men’s virtue signaling behaviors are often tied to their roles as resource providers and status seekers.

Moreover, the sexual selection of moral virtues provides another lens for understanding these differences. Traits like kindness, fidelity, and heroism may have evolved in both sexes through mutual mate choice, as they signal qualities such as reliability, cooperation, and parenting ability, which are critical for reproductive success and group cohesion (Fraser, 2010). However, the ways these traits are displayed often differ by sex. Women may emphasize traits that signal caregiving and social harmony, aligning with their traditional roles in nurturing and maintaining social bonds. In contrast, men may focus on traits that signal dominance, resource acquisition, and the ability to bear costs, reflecting their roles as providers and protectors (e.g., Buss, 1989).

Social norms further amplify these differences. Women are often expected to engage in daily, low-cost prosocial behaviors, such as caregiving and community involvement, which align with their traditional sex/gender roles (Eagly & Wood, 1991). Men, on the other hand, are more likely to engage in high-cost, high-visibility acts that demonstrate their ability to take risks and provide for others. These behaviors, such as physical risk-taking, are often linked to traits like bravery, competence, and social dominance, which align with their traditional sex/gender roles but are also valued in mate selection (Shan et al., 2012). Such acts not only enhance men’s status within their communities but also serve as signals of their genetic fitness and ability to protect and provide, making them more attractive as potential mates (Buss, 1989). Women’s focus on caregiving and social harmony may lead them to prioritize community-oriented and environmentally conscious behaviors, particularly when these actions are visible to others. In contrast, men’s emphasis on status and resource acquisition may drive them to engage in conspicuous, high-cost displays that signal their ability to bear costs and provide benefits to others. Thus, in the current study, we explore how sex differences manifest in self-reported propensity for virtue signaling related to environmentalism and community-mindedness. Specifically, we hypothesize both sexes will report that they would use environmentalism and community-mindedness to engage in virtue signaling and the benefits of virtue signaling should be equivalent. However, we hypothesize there will be measurable differences in the degree and context of virtue signaling between men and women. We predict that women may place greater importance on how others perceive their virtue signaling behaviors, congruent with evolutionary theories emphasizing the role of social reputation and cooperative alliances in female reproductive success. In contrast, while men also engage in virtue signaling, their behaviors may be more context dependent, such as in competitive or status-driven scenarios.

The rationale for this hypothesis is multifaceted. Women tend to be more environmentally and community-focused; for example, in Britain, an “eco-gender gap” exists whereby women are more likely to engage in behaviors such as recycling, turning down the thermostat, and decreasing water and food waste (Mintel, 2018). In addition, women value prosociality (e.g., Rand et al., 2016; Soutschek et al., 2017) and are often more attuned to social norms and expectations (Eagly & Wood, 1991), which can enhance their engagement in virtue signaling. These sex differences are also reflected in specific behaviors, such as ethical consumption. Women are often reported to value acting morally and behaving ethically more than men, which can translate into higher engagement in visible prosocial behaviors like recycling or purchasing eco-friendly products (Bateman & Valentine, 2010). Thus, we hypothesize that women will self-report a greater propensity to perform more conspicuous ethical consumption than men. That is, we predict women will purchase more ethical goods and do so for virtue signaling. However, men may be more likely to engage in public displays of environmentalism when these actions are tied to status or reputation, as seen in studies on conspicuous conservation (Griskevicius et al., 2010). We thus hypothesize (Hypothesis 1) that there will be a sex difference, with women scoring higher in how important others’ views of them are compared to men, but acknowledge that it holds importance to both sexes.

### Cross-Cultural Differences in Virtue Signaling

Cross-cultural differences in virtue signaling provide a valuable lens for understanding how individuals in different societies navigate social norms and express moral values. Take, for example, Japan, the USA, and Canada, which offer distinct cultural contexts that shape the motivations and expressions of virtue signaling behaviors. Japan, with its collectivist orientation and high long-term normative orientation (Hofstede Insights, 2021), emphasizes group harmony and social conformity. This cultural framework may lead individuals to engage in virtue signaling to avoid social disapproval and reinforce group cohesion, particularly in areas like environmentalism and community involvement. In contrast, the USA, characterized by high individualism and a consumer-driven economy, often associates virtue signaling with personal achievement and status enhancement (Amponsah & Owusuaa-Foster, 2025). Here, conspicuous ethical consumption and public displays of environmentalism may serve as signals of individual success and moral superiority. Canada, while also individualistic, reflects a more balanced approach, blending individualism with a collectivist ethos that prioritizes shared responsibility and environmental stewardship (Graf, 2024).

Recent research further addresses the role of social norms, trust, and institutional factors in shaping prosocial behaviors across cultures. For example, in societies with higher trust in institutions, individuals are more likely to engage in prosocial actions, such as environmental conservation or community involvement, as these behaviors align with societal expectations and norms (Graf, 2024). Similarly, conspicuous virtue signaling, such as publicly displaying eco-friendly behaviors, has been shown to enhance social status and influence green purchase intentions, particularly in cultures where social approval is highly valued (Amponsah & Owusuaa-Foster, 2025). We therefore also examined inter-country differences, given Japan is less individualistic (score 46) than Canada (score 80), which is similarly less individualistic than the USA (score 91; Hofstede Insights, 2021). Further, Japan has a higher normative long-term orientation (score 81) than Canada (score 36), which is higher than the USA (score 26; Hofstede Insights, 2021). Therefore, we predict as Hypothesis 2 that in Japan, self-reported propensity to virtue signal may be influenced by collectivist values and a focus on social conformity, leading individuals to behave in a manner that conform to community expectations. In the USA, individualism and status-driven norms may encourage virtue signaling as a means of personal achievement and social distinction, while Canada, with its blend of individualism and collectivism, may show individuals reporting a great propensity to virtue signal for behaviors tied to environmentalism and community-mindedness.

Sex differences in virtue signaling behaviors also vary across these cultural contexts, shaped by societal norms and expectations. In Japan, traditional gender roles emphasize women’s responsibility for maintaining social harmony and environmental stewardship, potentially leading to higher levels of community-minded virtue signaling among women compared to men (Sugimoto, 2010). Conversely, in the USA, where individualism and status-seeking behaviors are more noticeable, men may engage more in conspicuous ethical consumption as a way to signal wealth and status, consistent with costly signaling theory (Griskevicius et al., 2010). Canada, with its more egalitarian gender norms, may exhibit smaller sex differences in self-reported propensity to engage in virtue signaling behaviors, as both men and women are encouraged to participate in community and environmental initiatives (Blais & Gidengil, 1991).

Thus, for Hypothesis 3, for Japan, we predict that the traditional gender roles may amplify women’s focus on social cohesion and community-oriented virtue signaling, while men may engage less in these behaviors. In Canada, with its more egalitarian norms, sex differences in virtue signaling may be less noticeable, while in the USA, men may engage more in conspicuous, status-driven virtue signaling, while women may still emphasize prosocial and community-focused behaviors.

### Big Five Personality Differences

Typically, women are reported to score higher than men in neuroticism, extroversion, agreeableness, and conscientiousness, while men score higher on openness than women (e.g., Costa et al., 2001; Vianello et al., 2013). These trait differences are predicted to influence the propensity of engaging in virtue signaling behaviors: higher agreeableness and conscientiousness in women are likely to facilitate greater engagement in prosocial and community-oriented signaling, as these characteristics align with sensitivity to social norms and a propensity for cooperative behaviors. Elevated neuroticism may also contribute to increased concern with reputation management and others’ perceptions, further enhancing virtue signaling among women. By contrast, higher openness in men may correlate with engagement in status-oriented or unconventional forms of virtue signaling, such as conspicuous ethical consumption or creative outputs that publicly signal moral or environmental commitment.

Big Five personality traits are known to vary across cultures, too (Schmitt et al., 2008). For example, collectivist societies, such as Japan, tend to emphasize traits like agreeableness and conscientiousness, as these align with cultural values of group harmony, social cohesion, and adherence to societal norms (Markus & Kitayama, 1991; Nisbett, 2003). In such contexts, individuals with higher levels of these traits may be more likely to engage in virtue signaling behaviors that reinforce group values and demonstrate commitment to collective goals. In comparison, individualistic cultures, such as the USA, often place greater value on traits like openness and extraversion, which are associated with self-expression, innovation, and status-seeking (Hofstede Insights, 2021). In these contexts, virtue signaling may take on a more self-expressive or competitive form, with individuals using conspicuous displays of ethical consumption or public declarations of moral commitment to differentiate themselves and gain social recognition.

Thus, we hypothesized that the known sex differences in the Big Five personality traits would be linked to self-reported propensity for virtue signaling, with cultural context further shaping the expression of these behaviors. In collectivist societies, the link between agreeableness, conscientiousness, and virtue signaling may be stronger due to societal emphasis on group harmony and conformity. In contrast, in more individualistic contexts, such as the USA, openness and extraversion may be important for status-driven or self-expressive virtue signaling behaviors.

## Current Study

We propose that these expected patterns in self-reported propensity for virtue signaling behaviors reflect sex-based differences and the broader cultural frameworks in which they occur. Hence, the current study investigates how one’s propensity for virtue signaling behaviors as related to environmentalism and community-mindedness differ across Japan, the USA, and Canada. Specifically, we explore whether individuals in these countries self-report virtue signaling to enhance their social image, whether conspicuous ethical consumption varies by sex and culture, and how personality traits like the Big Five influence these behaviors. We test the three hypotheses in a sample of community participants in Canada, Japan, and the USA (*N* = 20,423).

To recap, then, we hypothesize the following:

1. Both sexes will report using environmentalism and community-mindedness to engage in virtue signaling, and the functional benefits should be equivalent across sexes.
2. Women will self-report a greater propensity to engage in conspicuous ethical consumption than men, consistent with theories of sex differences in social norm sensitivity and prosociality.
3. Sex differences in the Big Five personality traits will be associated with the tendency for virtue signaling, with cultural context further shaping these relationships.

## Method

### Participants and Procedure

We conducted an online survey in Canada, the USA, and Japan. We designed the questionnaires and outsourced their distribution and collection to an online survey company. The survey for the current study was included in a larger battery that pertained to nudging attitudes toward air pollution versus industrialization. The larger project investigated nudging effects designed to moderate citizens’ risk-averse attitudes. The topics included the trade-off between industrialization and air pollution. Conducted in the same three countries (Canada, Japan, and the USA), the studies demonstrated that messages emphasizing the support of older generations for industrialization reduced participants’ risk-averse attitudes toward industrialization (Komatsu et al., 2022). In the current studies, respondents were individuals who had registered with the company to participate in online surveys. The company obtained informed consent from all the participants on our behalf. While we did collect basic demographic information, the survey was anonymized and did not collect any personal information. Institutional ethics boards reviewed the research conducted per the Declaration of Helsinki and its later amendments.

Our analysis was based on samples from three countries (Table 1): samples obtained from February 25 to 27, 2020, in Japan (Japan 2020); February 27 to March 12, 2020, in Canada (Canada 2020); and February 27 to March 12, 2020, in the USA (USA 2020). For all the countries, the respondents were aged 20 years or older. Each respondent’s characteristics, including age and sex, were obtained from the survey company’s records. Each dataset consisted of equal numbers of male and female respondents, with proportional sampling according to each prefecture (Japan), province (Canada), and state (USA).

**Table 1.** Basic Attributes of the Dataset.

Dataset		Canada 2020	USA 2020	Japan 2020
Survey term		February 27–March 12, 2020	February 27–March 12, 2020	February 25–27, 2020
Number of samples	Male	3402	3405	3407
	Female	3403	3403	3403
Sex ratio (%)		100.0	100.0	100.1

**Figure 1.**
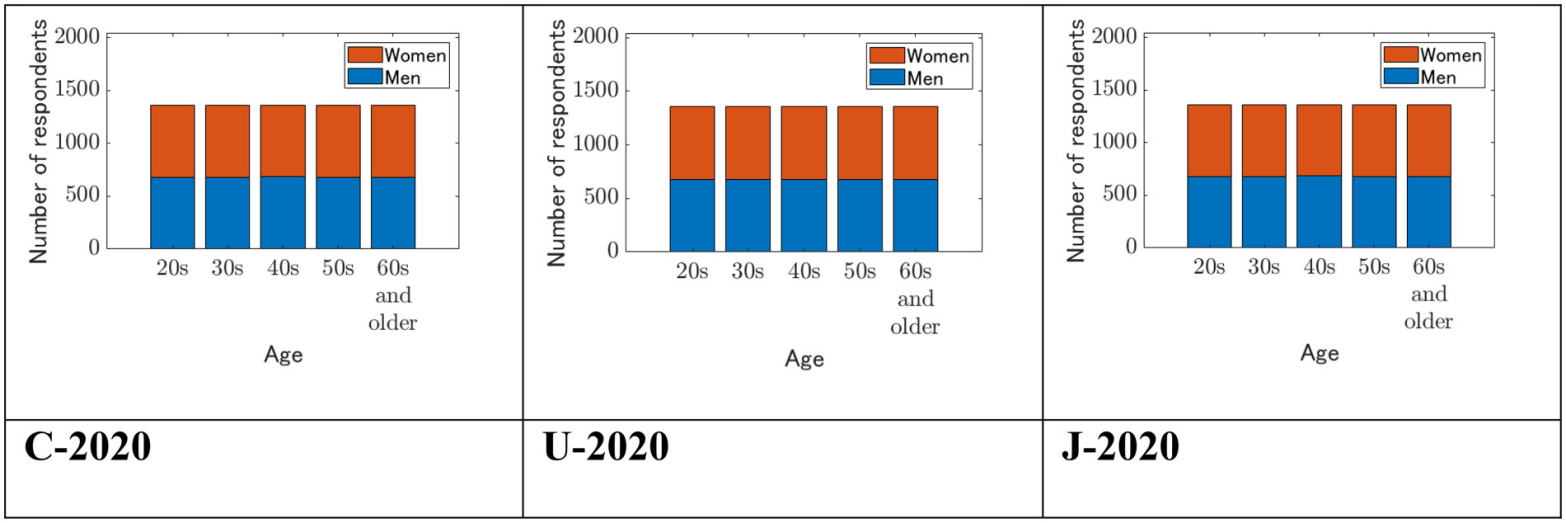
Number of respondents by age and sex.

The questionnaires were available in English and French for Canadian respondents, in English for US respondents, and in Japanese for respondents in Japan. The total number of valid samples for each dataset and sex ratios is shown in Table 1. The samples were collected with equal responses in the age groups of 20s, 30s, 40s, 50s, and 60s and older (see Figure 1).

### Measures

The items used in this study were developed based on existing literature that explores virtue signaling, ethical consumption, personality traits, and community-mindedness in the context of environmentalism and prosocial behaviors. For example, the questions related to ethical conspicuous consumption were inspired by research on green consumption values and the signaling of moral commitment through environmentally friendly products (Haws et al., 2014). Similarly, the virtue signaling items were informed by studies examining the social motivations behind pro-environmental behaviors and the desire to be perceived positively by others (Levy, 2020; Raihani & Bshary, 2015a, 2015b). The Big Five personality trait items were adapted to examine environmental and community-oriented behaviors, drawing on prior work linking personality traits to prosocial and environmentally conscious actions (Costa et al., 2001; Schmitt et al., 2008). Finally, the community-mindedness items were informed by research on them relating to pro-environmental behaviors and their connection to broader environmental factors (Koo, 2000).

Using this existing literature as a springboard, we measured virtue signaling using 5-point Likert-type scales (1 = *strongly disagree*, 5 = *strongly agree*) with three questions: “Being viewed positively by others is important to me” (which we hereon label as G1), “I try to be community-minded and act in an environmentally sensitive way because if I fail to do so, others will accuse me of not doing my part” (G2), and “I like others to see me engaged in activities where I seem environmentally conscious or community-minded” (G3). These items had an acceptable level of reliability (see Ursachi et al., 2015, for interpretation) with Cronbach’s *α* = 0.63 (0.63 for Canada, 0.59 for Japan, and 0.66 for the USA). The item-scale correlations (G1: 0.42, G2: 0.39, G3: 0.50) suggest that all three scales sufficiently contributed to the internal consistency and the moderate value of Cronbach’s *α* was likely due to the small number of items. The virtue signaling scale items (G1, G2, G3) were analyzed separately to capture distinctions between general importance of others’ views (G1) and specific motivations related to environmental and community actions (G2 and G3). This approach allows for a more detailed understanding of the different facets of virtue signaling. Note that while analyzing the items separately increases the number of comparisons, this approach was chosen to preserve the theoretical distinctions between the dimensions of virtue signaling. The results should be interpreted with caution to account for the potential for Type 1 errors.

There is no existing measure of ethical conspicuous consumption, so we created items that were measured on a 5-point Likert-type scale (1 = *strongly disagree*, 5 = *strongly agree*). These are “I like to wear clothes or show that I own items that reflect that I am a good person with strong morals” (C1), “It's important to me that others see me as wearing clothes or owning items that reflect my dedication to ethical or environmental protection” (C2), and “I try to buy clothes and items that are ethical or environmentally sensitive because it will increase how much other people like me” (C3). Together, these items had good reliability (Ursachi et al., 2015) with Cronbach’s *α* = 0.83 (0.81 for Canada, 0.85 for Japan, and 0.82 for the USA). The item-scale correlations (C1: 0.67, C2: 0.74, C3: 0.65) suggest that all the items sufficiently contributed to the internal consistency.

To examine the Big Five with virtue signaling, we created the following items based on the definition of each trait and then included application to environmentalism and community-mindedness. They were again measured on a 5-point Likert-type scale (1 = *strongly disagree*, 5 = *strongly agree*). The notation is provided here only for reporting purposes and was not seen by participants.

Openness (O): “I feel excitement and a high sense of enjoyment from doing activities that are community-oriented or for environmental protection.”

Conscientiousness (C): “I give my attention to my duties, which include being active in my community and engaging in behaviours that help protect the environment.”

Extraversion (E): “I have an excellent imagination and can see many ways to solve environmental problems or issues in my community.”

Agreeableness (A): “I do not like taking time out of my day to do something for the environment or my community.” (reverse-scored)

Neuroticism (N): “I never worry about things to do with the environment or my community.” (reverse-scored)

These items had an acceptable level of reliability, Cronbach’s *α* = 0.60 (0.62 for Canada, 0.47 for Japan, and 0.61 for the USA). Meanwhile, the item-scale correlations (O: 0.57, C: 0.54, E: 0.49, A: 0.20, N: 0.07) included low contributions to the internal consistency for A and N, which is consistent with known issues that reversed-score items often reduce the contributions. The Big Five personality trait items were adapted to examine environmental and community-oriented behaviors, rather than serving as a direct substitute for validated Big Five inventories. This approach was chosen due to the focus on how core personality traits are exhibited in specific prosocial domains, such as environmentalism and community-mindedness. While established Big Five measures provide a broad assessment of personality, our context-specific items were designed to capture the unique ways these traits are expressed in the targeted behavioral contexts. This decision reflects the aim to explore personality in relation to virtue signaling behaviors, rather than to provide a comprehensive assessment of general personality traits.

## Results

We used forced entry linear regression models by pooling the three datasets for 2020.

### Hypothesis 1

**Figure 2.**
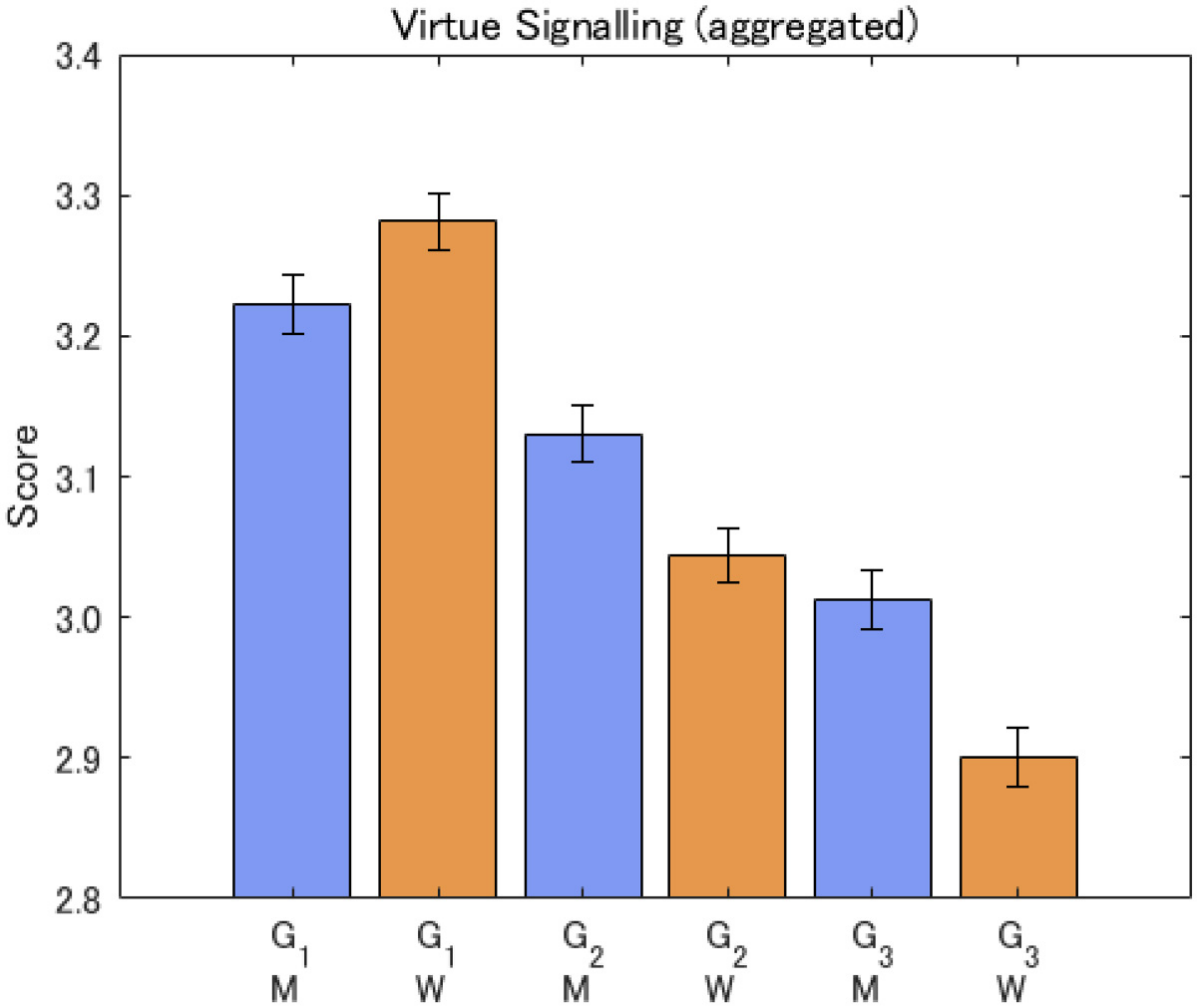
Responses to virtue signaling questions for aggregate sample. Note: The notation is M: men, W: women. G1, G2, and G3 refer to the items.

The importance of being viewed positively by others in a general sense was significantly higher for women than men (G1). However, once the questions focused on the more specific topics by mentioning community or environment, men reported significantly more importance of being viewed positively by others than women (G2 and G3). Also, more people were afraid of being accused of not being community-minded or environmentally sensitive rather than positively getting involved in community or environmental issues, as shown by the scores for G2 being larger than for G3. Refer to Figure 2.

**Figure 3.**
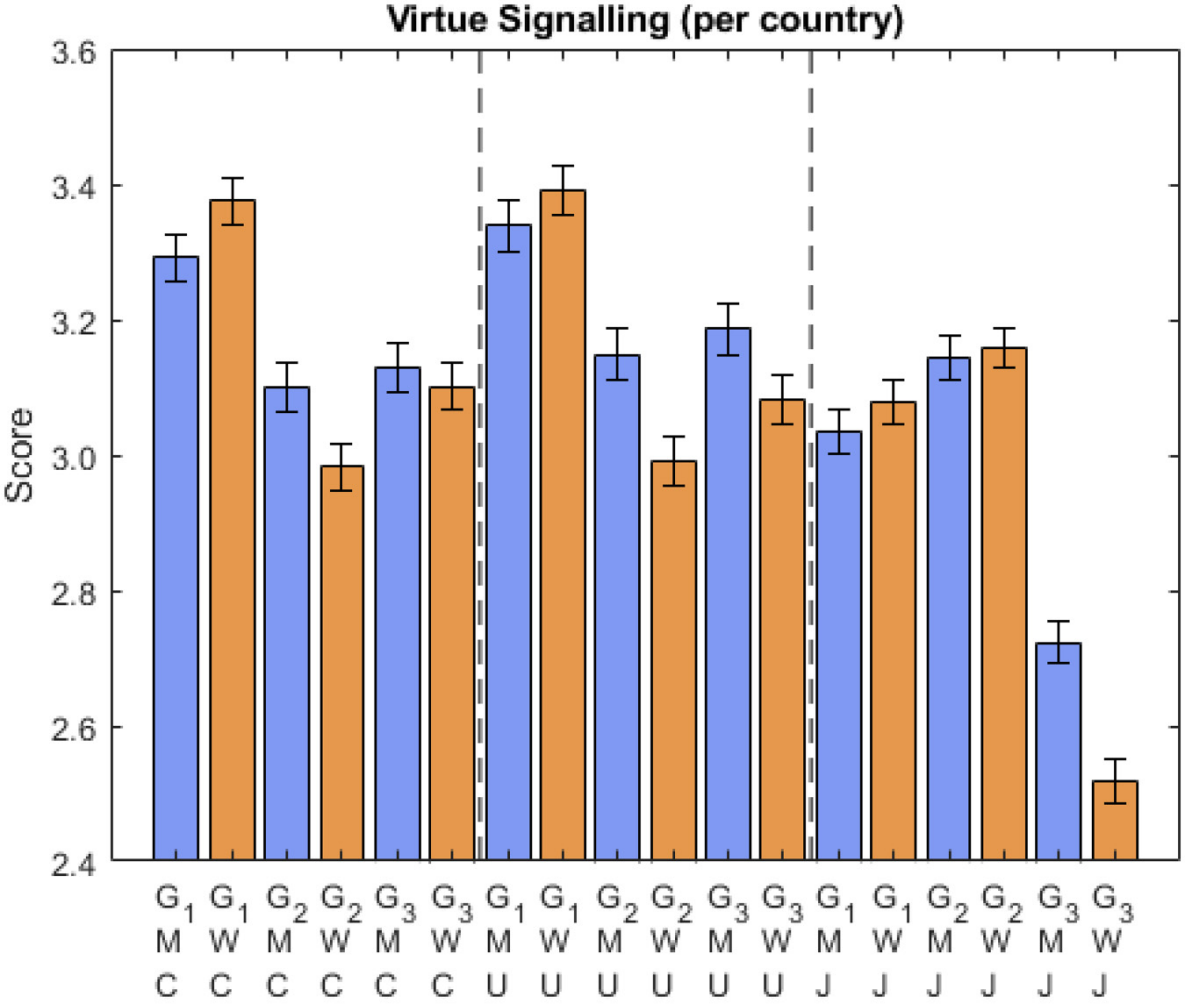
Responses about virtue signaling questions per country. Note: The notation is M: men, W: women, J: Japan, C: Canada, U: USA. G1, G2, and G3 refer to the items.

Regarding inter-country differences (Figure 3), men placed more importance on being actively viewed as environmentally sensitive and community-minded (i.e., they were actively willing to be viewed as so) in all three countries (G3), although scores were significantly lower in Japan. The importance of being passively viewed as environmentally conscious and community-minded (i.e., they were passively willing to be viewed as so because of fear of punishment) was stronger in men in Canada and the USA, too, with no significant sex difference in Japan (G2). Focusing on responses in Japan, the passive motivation (G2) was significantly higher than the active motivation (G3) and higher than for the other two countries. Age showed a significant negative contribution to each variable, suggesting that younger respondents are more sensitive to others’ views. While we did not have a specific hypothesis regarding age, previous research suggests that age can influence prosocial behaviors and social motivations, with younger individuals often prioritizing status-driven behaviors and older individuals focusing more on community-oriented actions (e.g., Griskevicius et al., 2010, p. 401). Including age allowed us to control for these potential effects and ensure that the observed relationships between sex, culture, and virtue signaling were not confounded by age-related differences. (Please refer to the Appendix for analyses with age removed, which are consistent with this main analysis.)

We used forced entry linear regression models by pooling the three datasets for 2020 (Japan 2020, Canada 2020, and USA 2020). Table 2 shows the results of the regression analysis.

**Table 2.** Coefficients from Linear Regression Analysis for Virtue Signaling.

Explained variables	G1–G3			G1			G2			G3		
	Estimated coefficients	SE	*T*	Estimated coefficients	SE	*T*	Estimated coefficients	SE	*T*	Estimated coefficients	SE	*T*
Intercept	3.51***	0.02	143.42	3.51***	0.03	108.59	3.45***	0.03	107.74	3.57***	0.03	109.01
Sex	−0.05***	0.01	−4.24	0.06***	0.01	4.10	−0.09***	0.01	−6.03	−0.11***	0.01	−7.67
Age	−0.07***	0.00	−17.89	−0.07***	0.01	−13.19	−0.07***	0.01	−13.78	−0.07***	0.01	−13.62
USA	0.03†	0.01	1.96	0.03†	0.02	1.75	0.03†	0.02	1.68	0.02	0.02	1.01
Japan	−0.22***	0.01	−16.44	−0.28***	0.02	−15.61	0.11***	0.02	6.27	−0.49***	0.02	−27.60
Adjusted *R*^2^	0.04			0.03			0.01			0.01		
Number of valid samples	20,423											

*Note*: ^†^ and *** indicate the difference from zero with 90% and 99.9% confidence, respectively. Sex coded 0 = men, 1 = women; USA and Japan coded 0 = no, 1 = yes; G1–G3 = mean of G1, G2, and G3.

### Hypothesis 2

**Figure 4.**
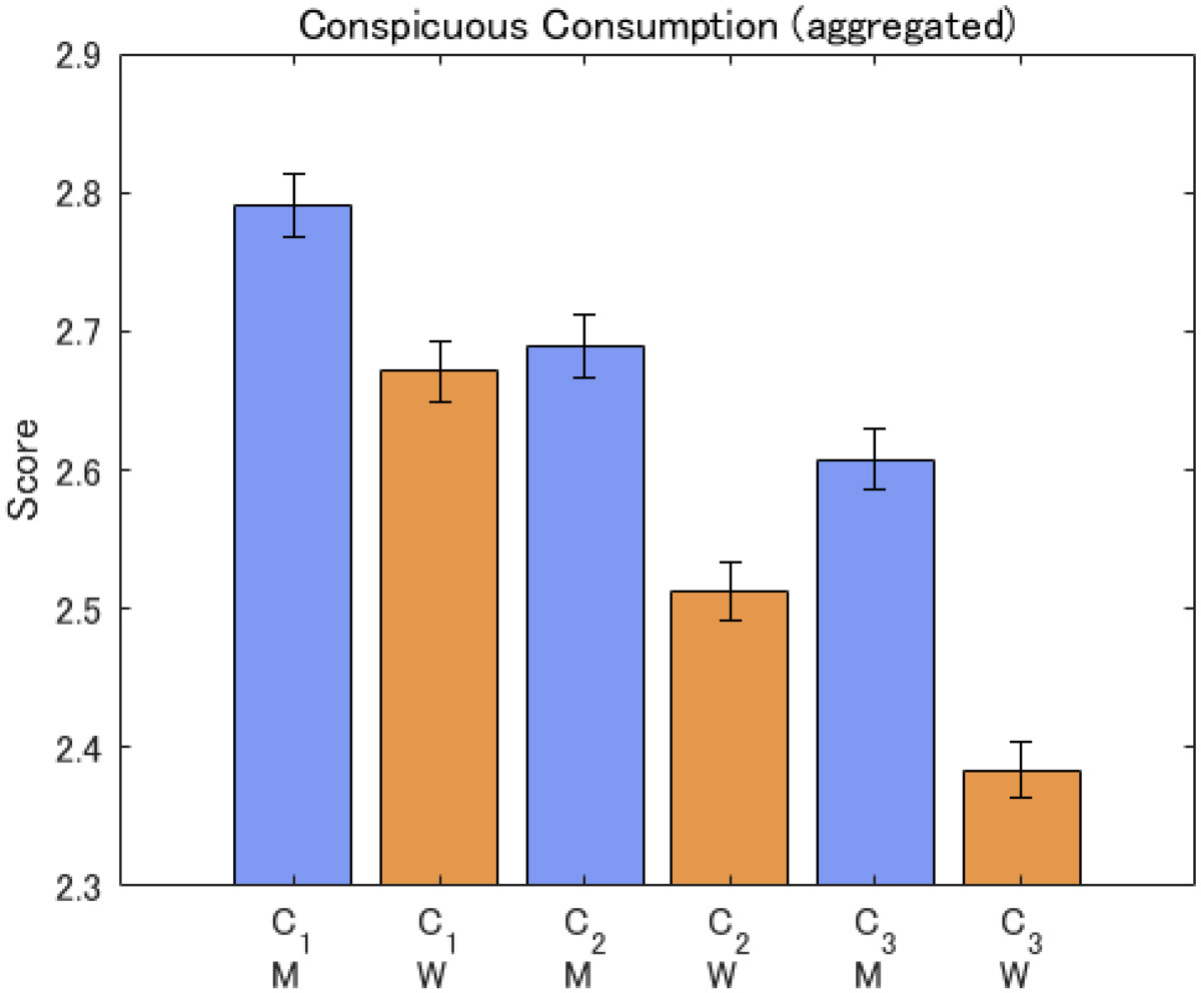
Responses to conspicuous consumption questions for aggregated sample. Note: The notation is M: men, W: women. C1, C2, and C3 refer to the items.

Ethical conspicuous consumption was more apparent for women than for men (C1, C2, and C3). All average scores were smaller than 3, suggesting that interest in ethical conspicuous consumption was not very positive or strong. However, the sex differences for all the questions were significant (see Figure 4). We used forced entry linear regression models by pooling the three datasets for 2020 (Japan 2020, Canada 2020, and USA 2020). Table 3 shows the results of the regression analysis. Again, similar to Hypothesis 1, age showed a significant negative contribution to each variable, suggesting that younger respondents engage more in ethical conspicuous consumption.

**Table 3.** Coefficients from Linear Regression Analysis for Conspicuous Consumption.

Explained variables	C1–C3			C1			C2			C3		
	Estimated coefficients	SE	*t*	Estimated coefficients	SE	*t*	Estimated coefficients	SE	*t*	Estimated coefficients	SE	*t*
Intercept	3.40***	0.03	115.41	3.51***	0.03	101.24	3.35***	0.03	98.06	3.33***	0.03	97.48
Sex	−0.17***	0.01	−13.15	−0.12***	0.02	−7.70	−0.18***	0.02	−11.56	−0.22***	0.02	−14.60
Age	−0.12***	0.00	−24.76	−0.12***	0.01	−22.16	−0.11***	0.01	−19.81	−0.12***	0.01	−21.69
USA	0.07***	0.02	4.38	0.09***	0.02	4.72	0.06**	0.02	3.24	0.06***	0.02	3.30
Japan	−0.27***	0.02	−16.81	−0.42***	0.02	−22.27	−0.23***	0.02	−12.10	−0.16***	0.02	−8.77
Adjusted *R*^2^	0.06			0.04			0.04			0.06		
Number of valid samples	20,423											

*Note*: ** and *** indicate the difference from zero with 99% and 99.9% confidence, respectively. Sex coded 0 = men, 1 = women; USA and Japan coded 0 = no, 1 = yes; C1–C3 = mean of C1, C2, and C3.

**Figure 5.**
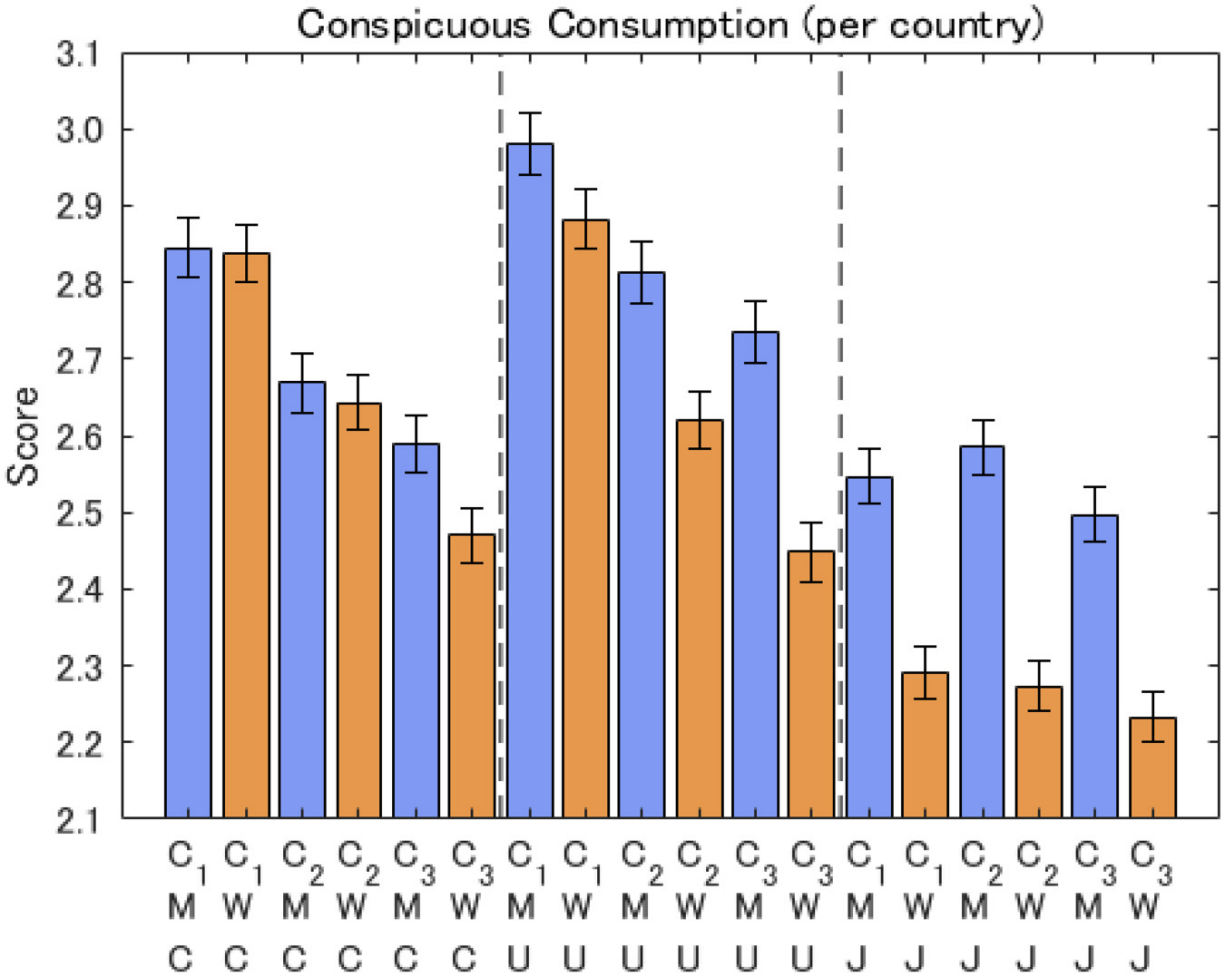
Responses about conspicuous consumption questions per country. Note: The notation is M: men, W: women, J: Japan, C: Canada, U: USA. C1, C2, and C3 refer to the items.

In terms of inter-country differences, the degree of conspicuous consumption averaged over all the questions was, in order, the USA > Canada > Japan (see Figure 5). The sex differences were the largest in Japan and the smallest in Canada (C1, C2, and C3).

### Hypothesis 3

Contrary to our hypothesis, there was no significant sex difference in openness. Also, while women showed higher scores in agreeableness and neuroticism, men showed higher scores in conscientiousness and extraversion. We used forced entry linear regression models by pooling the three datasets for 2020 (Japan 2020, Canada 2020, and USA 2020). Table 4 shows the results of the regression analysis. While age significantly showed a negative contribution to openness and extraversion, the contribution was positive for agreeableness and neuroticism.

**Table 4.** Coefficients from linear regression analysis for Big Five and Virtue Signaling.

Explained variables	OCEAN			O			C			E			A			N		
	Est coeff	SE	*t*	Est coeff	SE	*t*	Est coeff	SE	*t*	Est coeff	SE	*t*	Est coeff	SE	*t*	Est coeff	SE	*t*
Intercept	3.40***	0.02	174.13	3.81***	0.03	121.94	3.42***	0.03	112.20	3.74***	0.03	118.57	2.81***	0.03	86.30	2.96***	0.03	90.74
Sex	−0.01	0.01	−1.63	−0.02	0.01	−1.17	−0.07***	0.01	−5.09	−0.24***	0.01	−17.25	0.18***	0.01	12.62	0.19***	0.01	13.25
Age	−0.04***	0.00	−12.56	−0.08***	0.00	−16.50	0.01	0.00	1.62	−0.07***	0.00	−13.53	0.08***	0.01	16.03	0.07***	0.01	13.31
USA	0.01	0.01	0.56	0.05**	0.02	2.97	0.00	0.02	0.14	0.11***	0.02	6.10	−0.07***	0.02	−4.12	−0.15***	0.02	−8.19
Japan	−0.43***	0.01	−40.31	−0.76***	0.02	−44.73	−0.36***	0.02	−21.49	−0.54***	0.02	−30.97	−0.71***	0.02	−40.02	−0.33***	0.02	−18.76
Adjusted *R*^2^	0.10			0.13			0.03			0.09			0.10			0.03		
Number of valid samples	20,423																	

*Note*: Est coeff refers to estimated coefficients. ** and *** indicate the difference from zero with 99% and 99.9% confidence, respectively. Sex coded 0 = men, 1 = women; USA and Japan coded 0 = no, 1 = yes; OCEAN = mean of O, C, E, A, and N.

**Figure 6.**
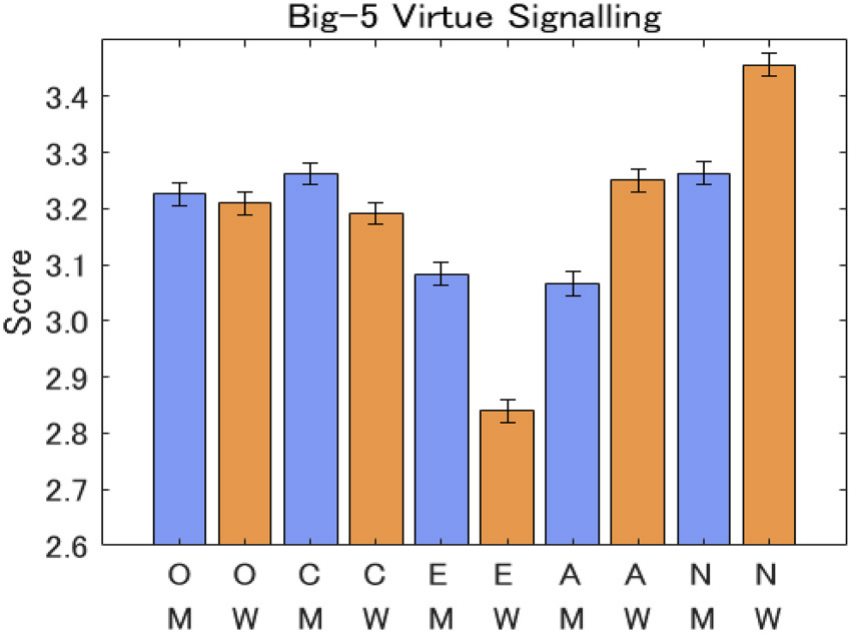
Responses to questions for Big Five and virtue signaling aggregate sample. Note: The notation is M: men, W: women, O: openness, C: conscientiousness, E: extraversion, A: agreeableness, and N: neuroticism.

**Figure 7.**
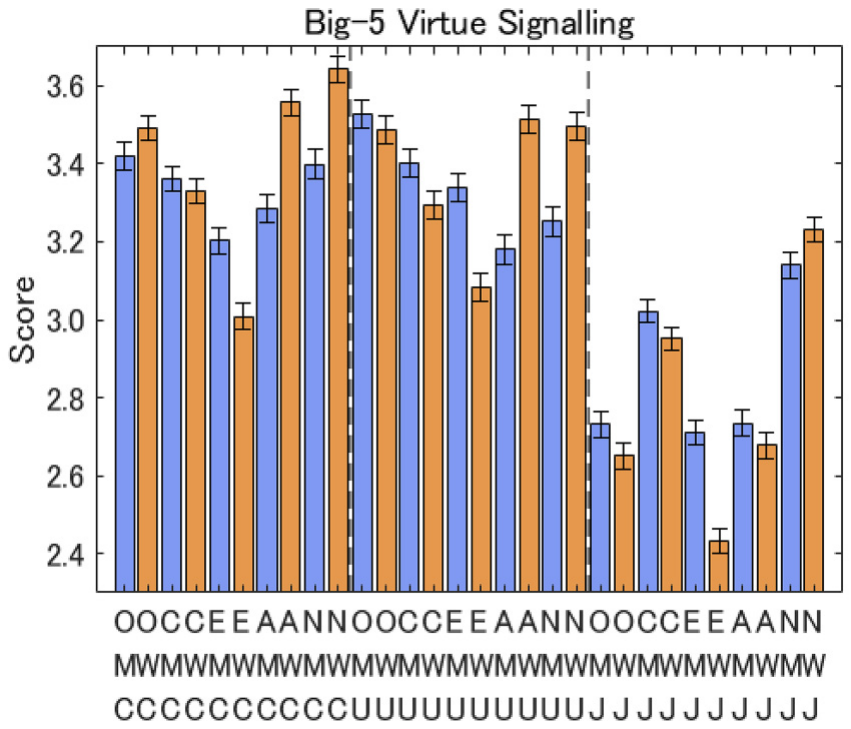
Responses to questions for Big Five and virtue signaling per country. Note: The notation is M: men, W: women, C: Canada, U: USA, J: Japan, O: openness, C: conscientiousness, E: extraversion, A: agreeableness, and N: neuroticism.

Regarding inter-country differences, all five items relating to the Big Five traits and virtue signaling were smaller in Japan than in Canada and the USA (see Figure 7). Further, while women showed higher openness than men in Canada, men showed higher openness than women in Japan, which resulted in the aggregated openness showing no significant sex difference, as shown in Figure 6. Please refer to the Appendix for further analyses.

## Discussion

Our goal was to investigate the virtue signaling of environmentalism and community-minded messages using evolutionary psychology. Virtue signaling spans many relevant topics well studied in this area, such as group dynamics, status and dominance, kinship, and mate choice, which often demonstrate significant differences for men and women. Virtue signaling behaviors, as observed in this study, fall under costly signaling theory, which suggests that individuals engage in resource-intensive or effortful behaviors to signal their fitness and value to others (Zahavi, 1975). For example, men’s higher engagement in conspicuous ethical consumption may reflect their use of status-driven signals to demonstrate resource acquisition and social dominance, traits that are desirable in mate selection (Miller, 2007). Women’s greater concern with being viewed positively by others, particularly in community-oriented contexts, may reflect their evolutionary role in promoting social cohesion and caregiving (Eagly & Wood, 1991).

Here, we explored sex differences by testing three hypotheses in community samples (*N* = 20,423) obtained in 2020 from Canada, Japan, and the USA. We hypothesized that both sexes use environmentalism and community-mindedness to engage in virtue signaling, which was supported by aggregate data across the countries. As we predicted, one’s interest in being viewed positively by others was significantly higher for women than for men. This finding is largely supported by evidence that women’s social image is highly important, possibly because they are more communal or rely on others more than men (e.g., Turner & Turner, 1999).

However, the situation becomes more complex when the individual items are examined. Once the questions focused on more specific topics by mentioning community or environment, men showed significantly more desire to be viewed positively by others, which was not expected. The reasons for this outcome warrant future study. Griskevicius et al. (2010) proposed that although men are typically more concerned about status striving and attempt to “show off” than women, they did not find sex differences in motivating status and when asked about consuming environmental products. They conjectured that maybe “men are more likely to engage in pro-environmental show-off displays, possibly because status motives may be more chronically active in men than in women. Nevertheless, women also strive for status, and our studies suggest that one status-striving tactic women use is displaying prosociality” (p. 401). Our findings support this possibility, but more work is needed.

While both sexes engage in status signaling, the underlying motivations and target audiences may diverge. For women, status signaling is often directed at other women as a tactic in intrasexual competition. Take, for example, Hudders et al. (2014) who found that women use luxury consumption to signal status to potential mates *and* deter same-sex rivals. In competitive mate-acquisition contexts, women show a higher preference for luxury items that enhance physical attractiveness, suggesting these items are used as a self-promotion strategy to gain an advantage over competitors. This finding is consistent with costly signaling theory, where luxury goods serve as reliable indicators of an individual’s value. The signal, however, has several dimensions. For women, it may communicate not only access to resources but also traits such as ambition, sexiness, and higher mate value to other women (Hudders et al., 2014). Thus, while men’s conspicuous displays may be more directly aimed at attracting mates by signaling resource-holding potential, women’s signaling may serve a dual purpose: attracting desirable mates while simultaneously managing and deterring intrasexual rivals. This possibility adds to the function of virtue signaling and conspicuous consumption in women, suggesting that the audience for the signal is potentially as important as the signal itself.

Also, when performing this analysis, we found that more respondents were afraid of being accused of not being community-minded or environmentally sensitive rather than positively getting involved in community or environmental issues. This finding may lead to the conclusion that people have greater concern about appearing as though they are not abiding by a social expectation or norm (i.e., others’ perceptions of oneself) rather than fulfilling a general interest. In many ways, this is the heart of virtue signaling; we originally defined it as one’s morals to improve their status and perceived value. This finding supports that definition. Thus, we note that the decision to analyze the virtue signaling scale items separately reflects the theoretical importance of distinguishing between general and specific dimensions of virtue signaling. While this approach increases the number of comparisons, it provides valuable information about the ways individuals engage in virtue signaling.

In Hypothesis 1, we additionally discovered that the desire to be viewed as environmentally conscious and community-minded in a passive way was stronger in Japan than in Canada and the USA when they are afraid of being accused of not getting involved. Preliminary research has shown that Japan generally emphasizes collective attitudes toward nature more than Canada, with societal norms and values emphasizing group harmony and environmental stewardship (Blaviesciunaite, 2012).

Second, we hypothesized that conspicuous ethical consumption, as a form of virtue signaling, would be performed more by women than men, which was not supported. Instead, significantly more men than women reported owning or wearing items displaying good morals or environmental awareness. All the average scores were small, although the sex differences for all items were significant. Similar to the points raised for Hypothesis 1, this finding may be better understood by costly signaling theory than virtue signaling, which suggests that such displays serve as signals of social status and wealth. According to this theory, an individual with luxury items displays to others that they can “waste” money and, consequently, signal that they are wealthy and have high social status (see for a review Berger, 2017; see also Hudders et al., 2014). The lack of support for our hypothesis that women engage more in conspicuous ethical consumption than men challenges traditional assumptions about sex differences in prosocial behaviors. Instead, our findings suggest that men may use conspicuous consumption as a costly signal of their ability to bear resources and provide benefits to others, congruent with their evolutionary role as resource providers (Buss, 1989; Griskevicius et al., 2010). This interpretation needs to be empirically tested, but it brings attention to the need to consider sexual selection pressures (and intrasexual competition specifically) in understanding the adaptive functions of virtue signaling.

Conspicuous environmentally friendly behaviors that others may readily observe may be at least partly explained by costly signaling theory (Griskevicius et al., 2012), leading to “green status” (Whitfield, 2011). Berger (2017) provides the example of a Toyota Prius, a hybrid car known for being less comfortable but at the same price as fuel-only models. Berger argues that the price is the part that makes the signal reliable, as one who truly cares about the environment, and hence others, will sacrifice comfort in this manner. While luxury and environmentally minded products both function to signal status, there is an important difference: luxury labels often enhance perceived status, and the latter may *also* signal prosocial values and cooperativeness, albeit without the same status impact (Berger, 2017). Therefore, environmentally-based signals may be intertwined with status more so than prosociality, making it more relevant to men than women (Griskevicius et al., 2010). This potential explanation needs further research attention.

We also documented inter-country differences, with conspicuous consumption being the highest in the USA, then Canada, and smallest in Japan. In the USA, high levels of conspicuous consumption are driven by individualistic cultural values and a consumer-oriented economy (as documented over a century ago by Veblen, 1899). Conversely, Canada’s conspicuous consumption, though present, is moderated by a more collectivist cultural orientation and regulatory measures that check consumer excess (Blais & Gidengil, 1991). Meanwhile, Japan displays more conservative consumption patterns with its cultural emphasis on modesty and social conformity, coupled with economic constraints like high living costs (Sugimoto, 2010).

Further, sex differences were the largest in Japan and the smallest in Canada. In Japan, traditional sex/gender roles are in play (Sugimoto, 2010) such that men are generally perceived as primary earners, and their consumption patterns may emphasize status, whereas women might prioritize practicality and sustainability in alignment with traditional caretaking roles (e.g., in fashion Kawamura, 2012; or more generally Sugihara & Katsurada, 2002). In contrast, a more egalitarian approach to sex/gender roles is seen in Canada, which diminishes disparities in consumer behavior between the sexes (Blais & Gidengil, 1991). The cultural emphasis, bolstered by government policy on gender equality and shared responsibility in environmental stewardship could lead to more homogeneous consumption patterns.

Third, we hypothesized that known sex differences in the Big Five personality traits are linked to virtue signaling, indicating how inherent personality differences may influence individuals’ behaviors that demonstrate moral virtues to others. Typically, studies have shown that women score higher than men in neuroticism, extraversion, agreeableness, and conscientiousness, while men often score higher in openness (Costa et al., 2001). These differences reflect evolutionary and socio-cultural roles, where women may prioritize social harmony and emotional expression, congruent with higher agreeableness and neuroticism (Schmitt et al., 2008). Our findings partially support this pattern. Contrary to previous research, no significant sex difference was observed in openness. Women showed higher scores in agreeableness and neuroticism, as predicted, which is congruent with traditional views of women as more empathetic and emotionally responsive (Costa et al., 2001) than men. Men, though, showed higher scores in conscientiousness and extraversion, which is not supported by past findings. The reasons for the difference in openness and conscientiousness deserve more investigation: it may be the case that the specific social dynamics and cultural expectations around community-mindedness and environmentalism may influence the expression of these traits and how they are observed in virtue signaling (Schmitt et al., 2008; 2009). A limitation of our current design is that the items for agreeableness and neuroticism showed lower contributions to the internal consistency than the other items. Future researchers may opt to refine the wording of these two items in particular.

All five factors were smaller in Japan than in Canada and the USA, which might be explained in terms of cultural orientations toward collectivism. In Japan, social harmony and group cohesion take precedence over individual expression, which may cause people to decrease their reporting of traits that emphasize individual distinction, such as extraversion and openness (Markus & Kitayama, 1991). Further, socio-cultural norms in Japan discourage displays of attributes such as extraversion and openness that are more freely expressed in the individualistic contexts of Canada and the USA (Nisbett, 2003). For example, self-effacement, where individuals downplay their abilities and characteristics in social settings, is prevalent in East Asian cultures (Heine et al., 1999).

There is much to be gained from studying virtue signaling using an evolutionary perspective. Signaling virtues may communicate one’s moral standards, enhancing social bonds and increasing cooperation (Smith & Bird, 2005). Virtue signaling can display a commitment to group norms and values, potentially making the individual more desirable as a cooperative partner or ally (Gintis, Smith & Bowles, 2001). Relatedly, they may be seen as prosocial, which could positively influence their social standing (Griskevicius et al., 2010). Signaling about environmental issues could be particularly useful in this way, as such messages are tightly linked to appearing cooperative with others (Barclay & Barker, 2020).

While these advantages are noteworthy and the current study provides valuable insights into the evolutionary underpinnings of virtue signaling, several limitations should be acknowledged, along with potential implications for future research. First, the reliance on self-reported data introduces the possibility of social desirability bias, particularly given the moral and prosocial nature of virtue signaling behaviors. Participants may have over-reported behaviors that are consistent with socially approved norms or under-reported behaviors perceived as self-serving. Future studies could address this limitation by incorporating observational methods or behavioral experiments to validate self-reported findings. For example, experimental paradigms that manipulate the visibility of virtue signaling behaviors (e.g., public vs. private settings) could reveal whether individuals are more likely to engage in these behaviors when they are being observed, as shown by Barclay and Barker (2020).

Second, the cross-sectional design of this study limits our ability to infer causality between personality traits, cultural contexts, and virtue signaling behaviors. Longitudinal studies could explore how virtue signaling behaviors develop over time and whether they are influenced by life history strategies, such as shifts in mating or parenting priorities. For instance, it is possible that younger individuals, who are more focused on mate acquisition, may engage in more conspicuous forms of virtue signaling, while older individuals may prioritize community-oriented behaviors that enhance group cohesion (Griskevicius et al., 2010).

Third, while we examined three cultural contexts (Canada, Japan, and the USA), the findings may not generalize to other cultures with different ecological or social pressures. For example, in cultures with high levels of resource scarcity, virtue signaling may take on different forms, such as signaling resource-sharing behaviors rather than conspicuous consumption. Similarly, in cultures with strong religious norms, virtue signaling may be more closely tied to adherence to religious practices rather than environmentalism or community-mindedness (Henrich, 2009). Expanding the scope of research to include a broader range of cultural settings would provide a more comprehensive understanding of how virtue signaling adapts to local ecological and social conditions.

Fourth, the measures used to assess virtue signaling and conspicuous ethical consumption were developed specifically for this study. While these measures demonstrated acceptable reliability, further refinement and validation are needed to ensure they fully capture the constructs of interest. For example, future research could explore whether the distinction between status-driven signaling and cooperation-driven signaling is reflected in different types of virtue signaling behaviors. It is possible that these two dimensions operate independently, with some individuals prioritizing status enhancement and others focusing on trust and cooperation (Barclay & Barker, 2020).

Last, this study did not directly measure the fitness benefits of virtue signaling behaviors, such as increased social status, mate attraction, or group cohesion. While the findings align with evolutionary theories, future research could test these hypotheses more directly. For instance, experimental studies could examine whether individuals who engage in conspicuous ethical consumption are perceived as more attractive or trustworthy by others, as suggested by Palomo-Vélez et al. (2020). Similarly, studies could investigate whether community-oriented virtue signaling enhances group cohesion and cooperation, particularly in high-stakes social dilemmas.

Outside of these limitations, there remain many directions for future work in addition to those already mentioned. For example, Heinrich (2009) argued that people engage in “credibility enhancing displays” (e.g., animal sacrifice and ritual mutilation) because actions speak louder than words. The cognitive processes associated with cultural learning allow learners to avoid being manipulated and to accurately assess one’s stated beliefs relative to their actions. If the display is costly, it is easy to determine whether the learner’s beliefs differ from what is expressed verbally. However, some signals are not costly but are credibility enhancing, such as facial expressions that are hard to fake yet not costly to produce and add credibility to one’s message. Virtue signaling may represent the same cognitive processes by learners in that one attempts to avoid manipulation. What is unique, though, is the potential benefit for the signaler of engaging in low-cost, hard-to-fake, credibility-enhancing displays to avoid being perceived as manipulating others for one’s own gain and, instead, seen as being genuine in their beliefs. Here, wearing pro-environmental clothing and driving an electric car may not be enough; looking sincerely physically pained at the sight of a new development plan resulting from a clear-cut forest might be more convincing. At the same time, a more general motivation to seek positive evaluation by others or simply to attract attention can exist alongside virtue signaling. Our current studies cannot guarantee that all of the items purely capture virtue signaling in a way that is clearly distinct from other such motivations. Future work should therefore examine ways to more clearly distinguish virtue signaling from related constructs. This step should also include refinement of the wording of items to more clearly distinguish community-mindedness from environmentalism, as these are conceptually related but potentially distinct constructs. Also, sex differences in virtue signaling may be better understood by better considering motives such as intrasexual competition or status-seeking, which could help clarify the core mechanisms involved.

Our findings suggest that virtue signaling serves both status signaling and social bonding functions, depending on the context and individual goals. Men’s higher engagement in conspicuous ethical consumption is congruent with costly signaling theory, as these behaviors may act as resource-intensive displays of fitness and status, enhancing their attractiveness as mates (Griskevicius et al., 2010; Zahavi, 1975). However, virtue signaling is not exclusively a form of status signaling. Women’s greater concern with being viewed positively in community-oriented contexts points to its role in promoting social cohesion and in-group trust, which are critical for group living and caregiving roles (Eagly & Wood, 1991). While virtue signaling and status signaling overlap, they are not synonymous; virtue signaling encompasses a broader range of behaviors, including those aimed at enhancing cooperation and trust. Cultural differences further indicate the adaptive flexibility of virtue signaling, with collectivist societies like Japan emphasizing group harmony and individualistic cultures like the USA prioritizing self-expression and status competition. These findings suggest virtue signaling has many distinct angles, presumably because it is an adaptive strategy for navigating social relationships.

This study explores virtue signaling of environmentalism and community-minded behaviors, revealing that both sexes self-report a propensity to engage in signaling across diverse cultural contexts. While the hypothesis that women more frequently engage in conspicuous ethical consumption than men was not supported, our research partially confirms the association between sex differences in the Big Five personality traits and virtue signaling behaviors. Our sample of community members in Canada, Japan, and the USA allowed us to examine the intercultural variations within these behaviors. These findings collectively point to the usefulness of studying virtue signaling from an evolutionary perspective, offering preliminary insight into its implications for individual strategies and the adaptive challenges of navigating social relationships in different cultural contexts.

## Appendix

[Table table5-14747049261423756] [Table table6-14747049261423756] [Table table7-14747049261423756] [Table table8-14747049261423756] [Table table9-14747049261423756] [Table table10-14747049261423756] [Table table11-14747049261423756] [Table table12-14747049261423756]

**Table A1. table5-14747049261423756:** Means, Standard Deviations, and Correlations Among Study Variables (*N* = 20,423).

	Mean	SD	Sex	Age	USA	Japan	G1–G3	C1–C3	OCEAN	G1	G2	G3	C1	C2	C3	O	C	E	A	N
Sex	1.50	0.50	1.00																	
Age	4.00	1.41	0.00	1.00																
USA	0.33	0.47	0.00	0.00	1.00															
Japan	0.33	0.47	0.00	0.00	−0.50	1.00														
G1–G3	3.10	0.80	−0.03	−0.12	0.08	−0.14	1.00													
C1–C3	2.61	0.97	−0.09	−0.17	0.10	−0.15	0.58	1.00												
OCEAN	3.08	0.66	−0.01	−0.08	0.16	−0.31	0.53	0.48	1.00											
G1	3.25	1.05	0.03	−0.09	0.08	−0.13	0.75	0.32	0.28	1.00										
G2	3.09	1.03	−0.04	−0.10	−0.01	0.04	0.72	0.48	0.39	0.28	1.00									
G3	2.96	1.08	−0.05	−0.09	0.12	−0.22	0.80	0.51	0.53	0.42	0.38	1.00								
C1	2.73	1.14	−0.05	−0.15	0.12	−0.19	0.51	0.86	0.40	0.32	0.38	0.45	1.00							
C2	2.60	1.11	−0.08	−0.14	0.07	−0.11	0.51	0.89	0.44	0.28	0.42	0.46	0.66	1.00						
C3	2.50	1.12	−0.10	−0.15	0.06	−0.08	0.48	0.84	0.41	0.24	0.44	0.42	0.55	0.64	1.00					
O	3.22	1.07	−0.01	−0.11	0.19	−0.35	0.47	0.44	0.77	0.27	0.35	0.44	0.38	0.41	0.36	1.00				
C	3.23	0.99	−0.04	0.01	0.09	−0.17	0.47	0.39	0.73	0.26	0.36	0.45	0.32	0.37	0.33	0.51	1.00			
E	2.96	1.06	−0.12	−0.09	0.17	−0.26	0.42	0.42	0.71	0.22	0.29	0.45	0.35	0.37	0.37	0.47	0.45	1.00		
A	3.16	1.10	0.08	0.11	0.12	−0.29	0.09	0.02	0.51	0.03	0.01	0.15	0.03	0.02	−0.01	0.30	0.24	0.18	1.00	
N	2.83	1.09	0.03	−0.18	−0.07	0.11	0.22	0.24	0.39	0.12	0.21	0.16	0.17	0.22	0.22	0.11	0.13	0.13	−0.16	1.00

*Note*: Sex coded 0 = men, 1 = women; USA and Japan coded 0 = no, 1 = yes; G1–G3 = mean of G1, G2, and G3; C1–C3 = mean of C1, C2, and C3; OCEAN = mean of O, C, E, A, and N; all correlations are Pearson's *r*.

**Table A2. table6-14747049261423756:** *p*-values for Correlations (*N* = 20,423).

	Sex	Age	USA	Japan	G1–G3	C1–C3	OCEAN	G1	G2	G3	C1	C2	C3	O	C	E	A	N
Sex	<0.001																	
Age	0.933	<0.001																
USA	0.996	0.986	<0.001															
Japan	0.972	0.986	<0.001	<0.001														
G1–G3	<0.001	<0.001	<0.001	<0.001	<0.001													
C1–C3	<0.001	<0.001	<0.001	<0.001	<0.001	<0.001												
OCEAN	0.123	<0.001	<0.001	<0.001	<0.001	<0.001	<0.001											
G1	<0.001	<0.001	<0.001	<0.001	<0.001	<0.001	<0.001	<0.001										
G2	<0.001	<0.001	0.095	<0.001	<0.001	<0.001	<0.001	<0.001	<0.001									
G3	<0.001	<0.001	<0.001	<0.001	<0.001	<0.001	<0.001	<0.001	<0.001	<0.001								
C1	<0.001	<0.001	<0.001	<0.001	<0.001	<0.001	<0.001	<0.001	<0.001	<0.001	<0.001							
C2	<0.001	<0.001	<0.001	<0.001	<0.001	<0.001	<0.001	<0.001	<0.001	<0.001	<0.001	<0.001						
C3	<0.001	<0.001	<0.001	<0.001	<0.001	<0.001	<0.001	<0.001	<0.001	<0.001	<0.001	<0.001	<0.001					
O	0.276	<0.001	<0.001	<0.001	<0.001	<0.001	<0.001	<0.001	<0.001	<0.001	<0.001	<0.001	<0.001	<0.001				
C	<0.001	0.112	<0.001	<0.001	<0.001	<0.001	<0.001	<0.001	<0.001	<0.001	<0.001	<0.001	<0.001	<0.001	<0.001			
E	<0.001	<0.001	<0.001	<0.001	<0.001	<0.001	<0.001	<0.001	<0.001	<0.001	<0.001	<0.001	<0.001	<0.001	<0.001	<0.001		
A	<0.001	<0.001	<0.001	<0.001	<0.001	0.013	<0.001	<0.001	0.049	<0.001	<0.001	0.004	0.149	<0.001	<0.001	<0.001	<0.001	
N	<0.001	<0.001	<0.001	<0.001	<0.001	<0.001	<0.001	<0.001	<0.001	<0.001	<0.001	<0.001	<0.001	<0.001	<0.001	<0.001	<0.001	<0.001

*Note*: Sex coded 0 = men, 1 = women; USA and Japan coded 0 = no, 1 = yes; G1–G3 = mean of G1, G2, and G3; C1–C3 = mean of C1, C2, and C3; OCEAN = mean of O, C, E, A, and N.

**Table A3. table7-14747049261423756:** Coefficients from Linear Regression Analysis for Virtue Signaling without Age Variable.

Explained variables	G1–G3			G1			G2			G3		
	Estimated coefficients	SE	*t*	Estimated coefficients	SE	*t*	Estimated coefficients	SE	*t*	Estimated coefficients	SE	*t*
Intercept	3.23***	0.02	169.20	3.24***	0.03	128.86	3.17***	0.02	127.23	3.28***	0.03	129.00
Sex	−0.05***	0.01	−4.22	0.06***	0.01	4.07	−0.09***	0.01	−6.01	−0.11***	0.01	−7.65
USA	0.03†	0.01	1.94	0.03†	0.02	1.74	0.03†	0.02	1.67	0.02	0.02	1.00
Japan	−0.22***	0.01	−16.32	−0.28***	0.02	−15.55	0.11***	0.02	6.24	−0.49***	0.02	−27.48
Adjusted *R*^2^	0.02			0.02			0.00			0.05		
Number of valid samples	20,423											

*Note*: † and *** indicate the difference from zero with 90% and 99.9% confidence, respectively. Sex coded 0 = men, 1 = women; USA and Japan coded 0 = no, 1 = yes; G1–G3 = mean of G1, G2, and G3.

**Table A4. table8-14747049261423756:** Coefficients from Linear Regression Analysis for Conspicuous Consumption without Age Variable.

Explained variables	C1–C3			C1			C2			C3		
	Estimated coefficients	SE	*t*	Estimated coefficients	SE	*t*	Estimated coefficients	SE	*t*	Estimated coefficients	SE	*t*
Intercept	2.94***	0.02	126.87	3.02***	0.03	111.27	2.92***	0.03	109.36	2.87***	0.03	106.89
Sex	−0.17***	0.01	−12.97	−0.12***	0.02	−7.62	−0.18***	0.02	−11.46	−0.22***	0.02	−14.45
USA	0.07***	0.02	4.31	0.09***	0.02	4.66	0.06**	0.02	3.20	0.06**	0.02	3.26
Japan	−0.27***	0.02	−16.57	−0.42***	0.02	−22.01	−0.23***	0.02	−11.99	−0.16***	0.02	−8.68
Adjusted *R*^2^	0.03			0.04			0.02			0.02		
Number of valid samples	20,423											

*Note*: ** and *** indicate the difference from zero with 99% and 99.9% confidence, respectively. Sex coded 0 = men, 1 = women; USA and Japan coded 0 = no, 1 = yes; C1–C3 = mean of C1, C2, and C3.

**Table A5. table9-14747049261423756:** Coefficients from Linear Regression Analysis for Big Five and Virtue Signaling without Age Variable.

Explained variables	OCEAN			O			C			E			A			N		
	Est coeff	SE	*t*	Est coeff	SE	*t*	Est coeff	SE	*t*	Est coeff	SE	*t*	Est coeff	SE	*t*	Est coeff	SE	*t*
Intercept	3.24***	0.02	213.68	3.48***	0.02	142.98	3.45***	0.02	146.14	3.47***	0.02	141.37	3.14***	0.03	123.70	2.67***	0.03	101.77
Sex	−0.01	0.01	−1.63	−0.02	0.01	−1.18	−0.07***	0.01	−5.09	−0.24***	0.01	−17.18	0.18***	0.01	12.55	0.07***	0.02	4.86
USA	0.01	0.01	0.55	0.05**	0.02	2.95	0.00	0.02	0.14	0.11***	0.02	6.07	−0.07***	0.02	−4.09	−0.06**	0.02	−2.98
Japan	−0.43***	0.01	−40.16	−0.76***	0.02	−44.44	−0.36***	0.02	−21.49	−0.54***	0.02	−30.83	−0.71***	0.02	−39.77	0.22***	0.02	11.81
Adjusted *R*^2^	0.10			0.12			0.03			0.08			0.09			0.01		
Number of valid samples	20,423																	

*Note*: Est coeff refers to estimated coefficients. ** and *** indicate the difference from zero with 99% and 99.9% confidence, respectively. Sex coded 0 = men, 1 = women; USA and Japan coded 0 = no, 1 = yes; OCEAN = mean of O, C, E, A, and N.

**Table A6. table10-14747049261423756:** Coefficients from Linear Regression Analysis for Virtue Signaling with Interaction Terms of Sex and Country Dummy Variable.

Explained variables	G1–G3			G1			G2			G3		
	Estimated coefficients	SE	*t*	Estimated coefficients	SE	*t*	Estimated coefficients	SE	*t*	Estimated coefficients	SE	*t*
Intercept	3.47***	0.03	102.85	3.48***	0.04	77.93	3.49***	0.04	79.23	3.44***	0.05	76.27
Sex	−0.02	0.02	−1.04	0.08**	0.03	3.35	−0.12***	0.02	−4.70	−0.03	0.03	−1.06
Age	−0.07***	0.00	−17.89	−0.07***	0.01	−13.19	−0.07***	0.01	−13.79	−0.07***	0.01	−13.63
USA	0.00	0.02	0.05	0.01	0.03	0.57	0.01	0.02	0.38	−0.02	0.03	−0.82
Japan	−0.24***	0.02	−12.40	−0.30***	0.03	−11.86	0.18***	0.02	7.08	−0.58***	0.03	−23.04
Sex*USA	0.05†	0.03	1.88	0.03	0.04	0.94	0.04	0.04	1.15	0.08*	0.04	2.17
Sex*Japan	0.03	0.03	1.10	0.04	0.04	1.16	−0.13***	0.04	−3.73	0.18***	0.04	4.97
Adjusted *R*^2^	0.04			0.03			0.01			0.06		
Number of valid samples	20,423											

*Note*: †, *, **, and *** indicate the difference from zero with 90%, 95%, 99%, and 99.9% confidence, respectively. Sex coded 0 = men, 1 = women; USA and Japan coded 0 = no, 1 = yes; G1–G3 = mean of G1, G2, and G3.

**Table A7. table11-14747049261423756:** Coefficients from Linear Regression Analysis for Conspicuous Consumption with Interaction Terms of Sex and Country Dummy Variable.

Explained variables	C1–C3			C1			C2			C3		
	Estimated coefficients	SE	*t*	Estimated coefficients	SE	*t*	Estimated coefficients	SE	*t*	Estimated coefficients	SE	*t*
Intercept	3.21***	0.04	79.27	3.34***	0.05	69.96	3.12***	0.05	66.41	3.18***	0.05	67.41
Sex	−0.05*	0.02	−2.20	−0.01	0.03	−0.23	−0.03	0.03	−0.96	−0.12***	0.03	−4.48
Age	−0.12***	0.00	−24.79	−0.12***	0.01	−22.18	−0.11***	0.01	−19.84	−0.12***	0.01	−21.71
USA	0.00	0.02	−0.02	0.04	0.03	1.64	−0.02	0.03	−0.86	−0.02	0.03	−0.85
Japan	−0.38***	0.02	−16.88	−0.55***	0.03	−20.39	−0.37***	0.03	−13.99	−0.24***	0.03	−8.95
Sex*USA	0.14***	0.03	4.41	0.09*	0.04	2.40	0.17***	0.04	4.45	0.17***	0.04	4.51
Sex*Japan	0.23***	0.03	7.04	0.25***	0.04	6.55	0.29***	0.04	7.67	0.15***	0.04	3.88
Adjusted *R*^2^	0.06			0.07			0.04			0.04		
Number of valid samples	20,423											

*Note*: *, **, and *** indicate the difference from zero with 95%, 99%, and 99.9% confidence, respectively. Sex coded 0 = men, 1 = women; USA and Japan coded 0 = no, 1 = yes; C1–C3 = mean of C1, C2, and C3.

**Table A8. table12-14747049261423756:** Coefficients from Linear Regression Analysis for Big Five and Virtue Signaling with Interaction Terms of Sex and Country Dummy Variable.

Explained variables	OCEAN			O			C			E			A			N		
	Est coeff	SE	*t*	Est coeff	SE	*t*	Est coeff	SE	*t*	Est coeff	SE	*t*	Est coeff	SE	*t*	Est coeff	SE	*t*
Intercept	3.29***	0.03	122.56	3.67***	0.04	85.40	3.36***	0.04	79.99	3.67***	0.04	84.33	2.68***	0.04	59.82	3.08***	0.05	67.20
Sex	0.06***	0.02	3.70	0.07**	0.02	2.99	−0.03	0.02	−1.28	−0.19***	0.02	−7.96	0.27***	0.03	10.86	0.16***	0.03	6.11
Age	−0.04***	0.00	−12.58	−0.08***	0.00	−16.50	0.01	0.00	1.62	−0.07***	0.00	−13.53	0.08***	0.01	16.08	−0.14***	0.01	−25.71
USA	−0.02	0.02	−1.36	−0.01	0.02	−0.23	−0.04	0.02	−1.53	0.07**	0.02	3.02	−0.04†	0.03	−1.70	−0.09***	0.03	−3.57
Japan	−0.51***	0.02	−33.74	−0.84***	0.02	−34.82	−0.38***	0.02	−16.06	−0.58***	0.02	−23.61	−0.88***	0.03	−34.94	0.13***	0.03	5.05
Sex*USA	0.05*	0.02	2.48	0.11**	0.03	3.30	0.08*	0.03	2.30	0.06†	0.03	1.83	−0.06†	0.04	−1.73	0.07*	0.04	2.02
Sex*Japan	0.16***	0.02	7.36	0.15***	0.03	4.49	0.04	0.03	1.23	0.08*	0.03	2.42	0.33***	0.04	9.26	0.18***	0.04	4.87
Adjusted *R*^2^	0.11			0.13			0.03			0.09			0.11			0.04		
Number of valid samples	20,423																	

*Note*: Est coeff refers to estimated coefficients. †, *, **, and *** indicate the difference from zero with 90%, 95%, 99%, and 99.9% confidence, respectively. Sex coded 0 = men, 1 = women; USA and Japan coded 0 = no, 1 = yes; OCEAN = mean of O, C, E, A, and N.
